# BumbleKey: an interactive key for the identification of bumblebees of Italy and Corsica (Hymenoptera, Apidae)

**DOI:** 10.3897/zookeys.784.25765

**Published:** 2018-09-12

**Authors:** Andree Cappellari, Maurizio Mei, Massimo Lopresti, Pierfilippo Cerretti

**Affiliations:** 1 Dipartimento di Biologia e Biotecnologie “Charles Darwin”, Università di Roma “La Sapienza”, Piazzale Aldo Moro 5, 00185, Rome, Italy; 2 CUTFAA, Raggruppamento Carabinieri Biodiversità, Via Carlo Ederle 16/A, 37100, Verona, Italy

**Keywords:** *
Bombus
*, bumblebee, identification tool, interactive key, Italy

## Abstract

BumbleKey is a matrix-based, interactive key to all 45 species of bumblebees of Italy and Corsica. The key allows to identify adult males and females (queens and workers) using morphological characters. The key is published online, open-access, at http://www.interactive-keys.eu/bumblekey/default.aspx.

## Introduction

The genus *Bombus* Latreille, 1802 includes around 260 species of eusocial bees, mostly distributed in temperate and cold regions of the northern hemisphere (Williams 2018). Of the 70 European species, 45 have been recorded from Italy and Corsica (Rasmont 1983, Intoppa et al. 1995, Rasmont and Adamski 1995, Rasmont and Iserbyt 2010–2013, Lecocq et al. 2015). Bumblebees are showy, large and loud insects, known to the public and easily recognizable as bees, and they are considered keystone species (Goulson 2010).

In the last decades, an apparent population decline of many species worldwide has been observed (Goulson et al. 2008, Williams and Osborne 2009, Goulson 2010, Rasmont et al. 2015). Even if the causes are still not clear, this decline is probably driven by a complex combination of factors like the reduction and fragmentation of natural habitats (especially for mountain species), climate changes and heat waves, use of pesticides, and reduction and alteration of floral resources. On the other hand, some species, the most generalist and heat tolerant ones, are expanding their ranges (Rasmont et al. 2015).

Bumblebees are pollinators of great ecological and commercial importance throughout much of the temperate world; the industry of greenhouse crop pollination is worth billions of dollars each year (Velthuis and Doorn 2006).

In Italy ecological studies focused on the effects of climate change and anthropogenic pressure on bumblebee communities, on their role as pollinators, or on the ecology of bumblebees in general, are very few. This is due, at least in part, to the difficulty in identifying these insects to species level by non-specialists. Moreover, the actual occurrence and the detailed distribution of bumblebee species in Italy are poorly known. In this respect, information is based on scattered, occasional, and often old or unreliable data that needs to be updated.

Surprisingly, however, bumblebees are difficult to identify to species level even for experienced taxonomists. They show great intraspecific variability in colouration pattern and a remarkable regional interspecific colouration pattern convergence, which is usually explained in terms of Müllerian mimicry (Plowright and Owen 1980, Williams 2007). Otherwise, they are morphologically monotonous showing only subtle differences in diagnostic character states (Michener 2007).

Available keys, all dichotomic, for the identification of West-Palaearctic bumblebees are hardly accessible for non-specialists, often lacking of adequate iconography supporting character states assessment (Pittioni 1939, Løken 1973, 1984, Amiet 1996, Intoppa et al. 2009, Gorcezade et al. 2010, Intoppa et al. 2014). BumbleKey, a matrix-based interactive key for the identification of Italian bumblebees, is intended to be a flexible, practical and easily accessible tool, that could be useful to increase the interest for these bees and to facilitate and foster different kind of studies (ecological, conservation, etc.).

## Project description

### Study area

**Figure 1.**
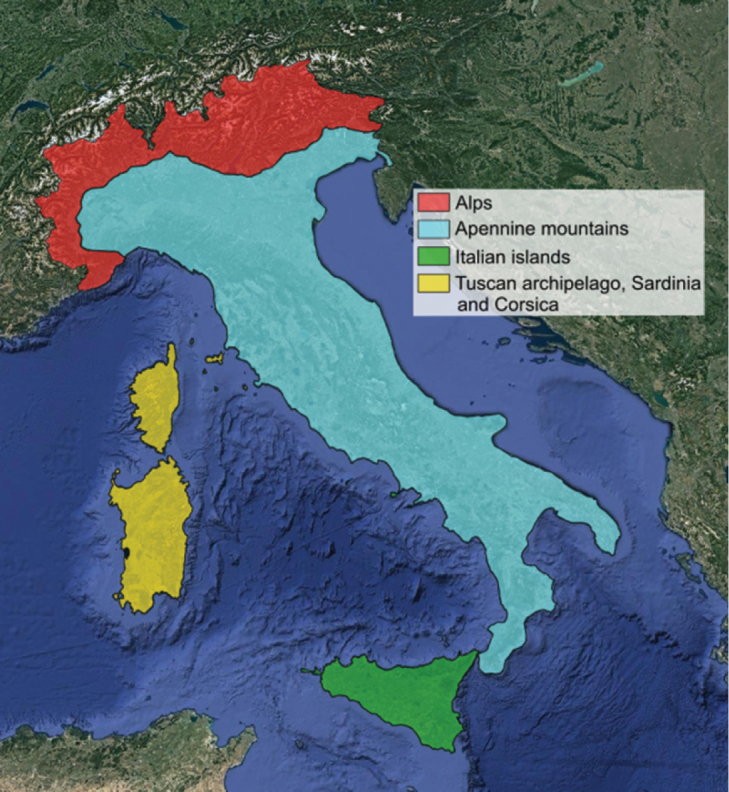
Map of the four regions in which the study area is divided.

The study area includes Italy (political boundaries) and Corsica. For practical reasons, the area has been divided into four regions: Alps, Apennine mountains, Italian islands (Sicily and minor islands), Tuscan Archipelago, Sardinia and Corsica (Fig. 1). Coordinates are between 35°28'56"N and 47°07'04"N Latitude, and between 8°12'08"E and 18°32'15"E Longitude.

### Taxonomic coverage

The key includes all the 45 species of bumblebees that have been recorded for the study area (Rasmont and Adamski 1995, Intoppa et al. 1995, 2009, Lecocq et al. 2015, Martinet et al. 2018). A few species, i. e. *Bombusconfusus* Schenck, *B.pomorum* (Panzer), *B.veteranus* (Fabricius), have not been collected in the last several decades and their presence in Italy is doubtful and should be confirmed by new records. The single published record of *B.magnus* Vogt and the two records of *B.distinguendus* Morawitz are based on misidentifications. *Bombusxanthopus* Kriechbaumer and *B.renardi* Radoszkowski, traditionally considered as Corsican subspecies of *B.terrestris* (Linnaeus) and *B.lucorum* (Linnaeus) respectively (Estoup et al. 1996, Williams et al. 2012, Lecocq et al. 2013, Williams 2018), are here treated at rank of species according to Lecocq et al. (2015).

### List of the terminal taxa included in the current version of the identification key (last update 10 April 2018)

*Bombusalpinus* (Linnaeus, 1758); *B.argillaceus* (Scopoli, 1763); *B.barbutellus* (Kirby, 1802); *B.bohemicus* Seidl, 1837; *B.brodmannicus* Vogt, 1909; *B.campestris* (Panzer, 1802); *B.confusus* Schenck, 1859; *B.cryptarum* (Fabricius, 1775); *B.distinguendus* Morawitz, 1869; *B.flavidus* Eversmann, 1852; *B.gerstaeckeri* Morawitz, 1882; *B.hortorum* (Linnaeus, 1761); *B.humilis* Illiger, 1806; *B.hypnorum* (Linnaeus, 1758); *B.inexspectatus* (Tkalcu, 1758); *B.jonellus* (Kirby, 1802); *B.konradini* Reinig, 1965; *B.lapidarius* (Linnaeus, 1758); *B.lucorum* (Linnaeus, 1761); *B.magnus* Vogt, 1911; *B.mendax* Gerstaecker, 1869; *B.mesomelas* Gerstaecker, 1869; *B.monticola* Smith, 1849; *B.mucidus* Gerstaecker, 1869; *B.muscorum* (Linnaeus, 1758); *B.norvegicus* (Sparre-Schneider, 1918); *B.pascuorum* (Scopoli, 1763); *B.pomorum* (Panzer, 1805); *B.pratorum* (Linnaeus, 1761); *B.pyrenaeus* (Pérez, 1879); *B.quadricolor* (Lepeletier, 1832); *B.renardi* Radoszkowski, 1884; *B.ruderarius* (Muller, 1776); *B.ruderatus* (Fabricius, 1775); *B.rupestris* (Fabricius, 1793); *B.sichelii* Radoszkowski, 1859; *B.soroeensis* (Fabricius, 1776); *B.subterraneus* (Linnaeus, 1758); *B.sylvarum* (Linnaeus, 1761); *B.sylvestris* (Lepeletier, 1832); *B.terrestris* (Linnaeus, 1758); *B.vestalis* (Geoffroy in Fourcroy, 1758); *B.veteranus* (Fabricius, 1793); *B.wurflenii* Radoszkowski, 1859; *B.xanthopus* Kriechbaumer, 1870

### Applicability and operational methods

The key is designed to allow species-level identification of adult bumblebee specimens, both males and females. The sex of the specimen must be assessed as a first step, as explained in the Instructions section of the key. Specimens should be pinned, and the male genitalia capsule must be pulled out from the metasoma. No dissection or further special preparation are required. All the characters can be examined by means of a stereomicroscope, with incident light and at convenient magnification. Some of the most obvious colour pattern traits can be even assessed to the naked eye.

Thanks to the non-hierarchic structure of the key, the identification of damaged specimens, or part of them, is to some extent possible.

### General features of characters used in the key

Among the characters traditionally employed in the relevant literature as diagnostic, the ones that are too difficult to observe or to evaluate, as well as those whose evaluation is too subjective or time consuming (e. g. complex measurements) have been excluded. All the characters and states have been verified and evaluated on a reference collection of specimens of all the 45 species included in the key. The reference specimens consist of males, queens, and workers of each species collected on purpose by the authors or preserved in the following collections: Museo di Zoologia, Sapienza Università di Roma, Rome, Italy (MZUR), Museo Civico di Zoologia, Rome, Italy, and M. Mei personal collection (Rome).

A total of 50 diagnostic characters have been selected, 10 relevant to both males and females, 25 relevant only to females, and 15 relevant only to males. 19 characters are binary, and 31 are multistate with 3-9 alternative states each, for a grand total of 185 states. No character is dependent on the state of another; therefore, their applicability is not constrained, the only limitation being the sex of the specimen to identify.

In many instances, especially in the colouration pattern section, more than one state has been assigned to a character for a given species, to cover most of the intraspecific variability or to avoid possible subjectivity in the assessment of certain character states.

For cryptic species, i. e. those in the so-called “*Bombuslucorum*-complex” (Bossert 2015), and for morphologically very similar species, it could be difficult or impracticable to end up a single taxon using the characters in the key. In such cases, additional diagnostic information is given in the relevant species files.

### List of the characters used in the key

PATTERN: face and clypeus hair colour; upperside of the head (vertex) hair colour; bands on mesosoma; mesopleurae main colour; wing colour; hind tibia cuticle colour; hind tibia hair colour; bands on metasoma; metasomal tergites 4-7main colour; metasomal tergites 4-7colour arrangement.

HEAD: [F] antenna, antennomeres A3-A5; [F] antenna – antennal segment A3 ratio; [M] antenna – median antennomeres (A5-A9); [M] eye; [F] ocello-ocular area, sculpture; [F] ocello-ocular area, unpunctured and shining areas; [F] oculo-malar area; [M] oculo-malar area; [F] clypeus, shape; [F] labrum, overall shape; [F] labrum, median furrow and lateral tubercles (overall shape); [F] labrum, median furrow; [F] labrum, lamella shape; [F] labrum, lamella dimension; [F] mandible, number of teeth; [F] mandible, shape; [F] mandible, anterior keel; [F] mandible, sulcus obliquus (posterior groove); [F] mandible, incisura (distal notch); [M] mandible, “beard”.

MESOSOMA: [F] mid basitarsus, distal posterior corner; [F] mid basitarsus, erect hairs on the outer surface; [F] hind tibia, outer surface; [M] hind tibia, outer surface; [F] hind tibia, corbicula; [M] hind tibia, posterior fringe hairs; [F] hind basitarsus, proximal posteriorly-directed process; [F] hind basitarsus: length of posterior fringe’s hairs.

METASOMA: [F] metasomal tergite 6 (T6); [F] metasomal sternite 2(S2); [F] metasomal sternite 6(S6), keels; [M] volsella and gonostylus; [M] volsella, dorsal view; [M] volsella, overall shape; [M] volsella, process of the inner margin; [M] gonostylus, dorsal view; [M] gonostylus, inner process; [M] penis valve, head, dorsal view; [M] penis valve, shaft; [M] gonocoxa, apex, dorsal view.

### Iconography

Every character state is illustrated by 1 to 4 pictures (214 in total). For each species, a minimum of 5 pictures (dorsal female and male habitus, frontal view of female and male head, and male genitalia) have been provided in the species window. For most species, however, multiple pictures (up to 16) are available, in order to illustrate the range of colour-pattern intraspecific diversity or important diagnostic morphological details. The morphological terminology used in the key is illustrated by original line drawings.

### Software technical specifications

*Platform*: Framework.Net

*Web Server*: Microsoft Internet Information Service 6.0

*Programming language*: C#

*Application version*: MOSCHweb 1.0

*Data base*: Microsoft SQL Server

*Data*: 1.0beta

*Language*: English

*License for use of the key*: Creative Commons Attribution License 3.0 (CC-BY), which permits unrestricted use, distribution, and reproduction in any medium, provided the original author and source are credited.

*Use of the primary data*: Primary data are available from the authors by agreement.

*Web Location*: http://www.interactive-keys.eu

### Software technical features

**Figure 2.**
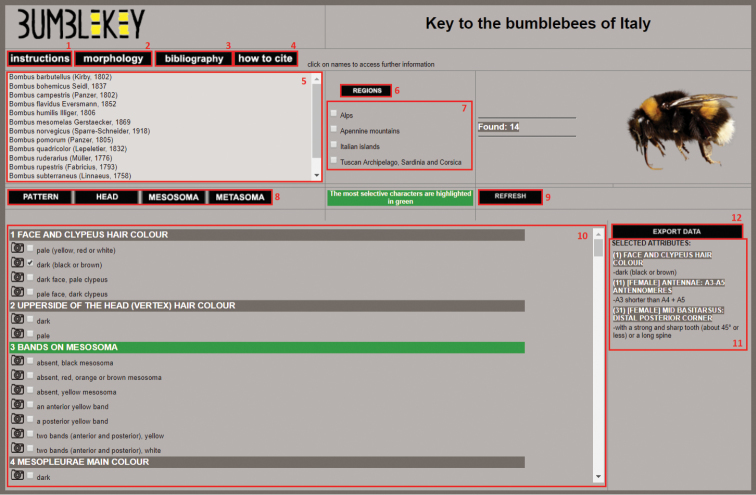
Interactive key main page.

BumbleKey interface is simple and intuitive (Fig. 2). The chosen software is MOSCH, originally developed for DipteraTachinidae, and later used for other groups (Cerretti et al. 2012, Bonato et al. 2014).

1 – *Instructions*: a button that refers to a dedicated window, where the user can find information about how to use the key.

**Figure 3.**
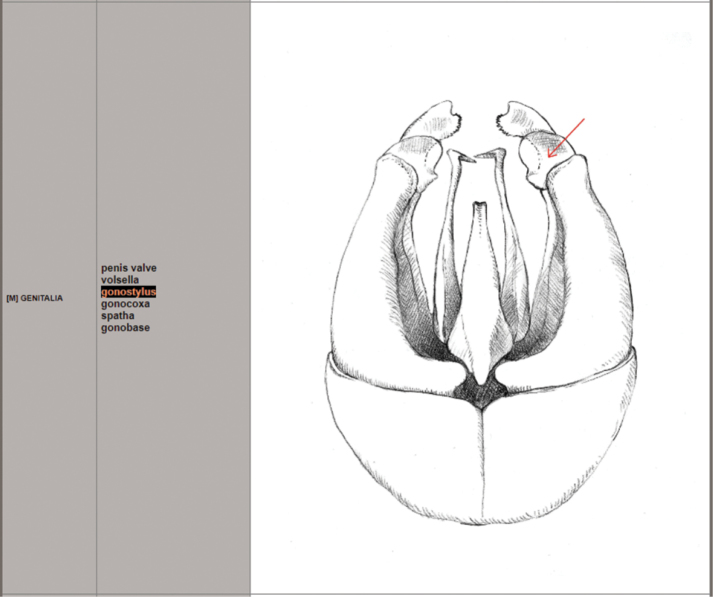
Interactive image window for morphology.

2 – *Morphology*: a button that refers to a dedicated window (Fig. 3), with drawings illustrating the terminology used in the key and pictures of different shades of hair colours. Images are obtainable just by hovering over entries.

3 – *Bibliography*: a button that refers to a dedicated window, illustrating a selection of papers used for the creation of the key.

4 – *to cite*: a button that refers to a dedicated window, illustrating how user should cite the key.

**Figure 4.**
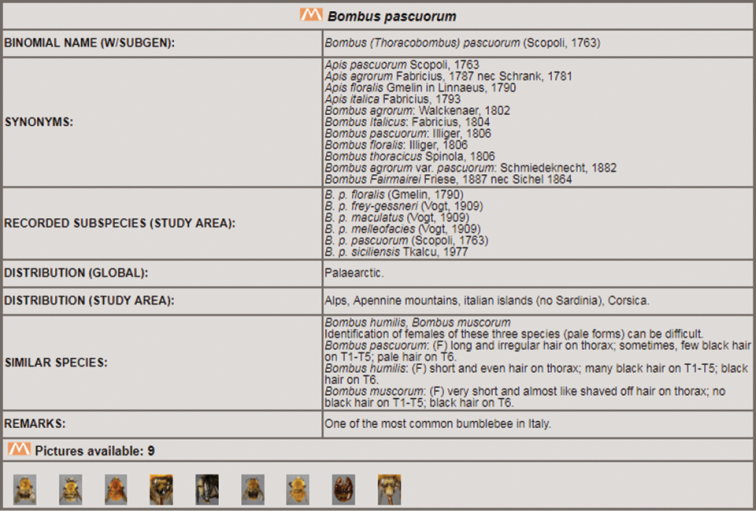
Example of taxon window.

5 – *Species window*: an updating real-time box showing all the species that share selected character states. The name of the species is followed by the author name and the year of description. Clicking on a species name, a new window opens to show more information about that species (Fig. 4). The *taxon window* shows binomial name and subgenus, main synonyms, recorded subspecies in the study area, distribution worldwide and in the study area, similar species with additional diagnostic characters, remarks, and pictures. Each species picture can be opened in a dedicated window, for example to be compared with other pictures, by clicking on its name in the *double temporary view* section.

6 – *Regions*: a button that refers to a dedicated window, showing a map of the four regions in which the study area is divided.

7 – *Regions menu*: the user can select the region of origin of the specimen to reduce the query to the taxa belonging to that region. By default, all the regions are selected.

8 – *Body parts bar*: a bar with buttons that allow the user to quickly reach the desired section. Characters are arranged in four sections: pattern (10 characters), head (20 characters), mesosoma (8 characters), and metasoma (12 characters).

9 – *Refresh*: a button that clears the checkboxes for characters and regions.

10 – *Character window*: a window with all the characters used in the key and pictures of all the states. Each state picture can be opened in a dedicated window, for example to be compared with other pictures, by clicking on its name in the *double temporary view* section. The characters can be used in any order, and the more selective ones are highlighted in green. BumbleKey allows for uncertainty to be expressed by the selection of more than one state for each character.

11 – *Selected choice box*: an updating real-time box showing the chosen characters and state selected by the user, ordered as they appear in the *Character window*. This represents an ID-code which is linked to the specimen under examination

12 – *Export data*: a button allowing the user to export in TXT format the terminal taxon/taxa, followed by the list of selected states in the form of a code, together with the specification of the used BumbleKey version. This code serves as a record of the character states used to achieve a specimen identification.

## Conclusions and future work

MOSCHweb is an open-access web application, but it is not open source. The application can be modified or updated only by, or in agreement with, the authors of this paper. The authors will keep updated both the web application and the data matrix, by improving encoded descriptions of terminal taxa or by adding possible new taxa to the matrix. Indeed, one of the future desirable developments of BumbleKey will be to better document the chromatic and morphological variation of every species and to extend the key to include all the European and possibly West-Palaearctic species. This would be very difficult with a traditional, dichotomous tool, but with MOSCHweb the problem would be easily solved by augmenting the database with the addition of new data strings to the matrix.

The taxonomy and systematics of Italian and European bumblebees are thoroughly studied and relatively stable (Cameron et al. 2007, Rasmont and Iserbyt 2010–2013). However, recent revisions and phylogeographic studies have led to several taxonomic changes (Rasmont et al. 2005, Williams et al. 2008, Lecocq et al. 2011, Lecocq et al. 2015, Martinet et al. 2018). Moreover, possible faunal changes can result from the increase of species ranges due to climate change (Neumayer 2004, Jenič et al. 2010, Šima and Smetana 2012, Martinet et al. 2015) or from the introduction of species or subspecies from other regions as a consequence of the commercialization of bumblebee colonies for pollination (Ings et al. 2010). When necessary, the key could be easily augmented or modified to cope with such minor changes too. Any modification will be reported at http://www.interactive-keys.eu.
